# Intranasal Oxytocin for Negative Symptoms of Schizophrenia: Systematic Review, Meta-Analysis, and Dose-Response Meta-Analysis of Randomized Controlled Trials

**DOI:** 10.1093/ijnp/pyab020

**Published:** 2021-04-23

**Authors:** Michel Sabe, Nan Zhao, Alessio Crippa, Gregory P Strauss, Stefan Kaiser

**Affiliations:** 1 Division of Adult Psychiatry, Department of Psychiatry, Geneva University Hospitals, Geneva, Switzerland; 2 Department of Public Health Sciences, Karolinska Institutet, Stockholm,Sweden; 3 Department of Psychology, University of Georgia, 125 Baldwin St., Athens, GA, 30602, USA

**Keywords:** Asociality, intranasal oxytocin, negative symptoms, oxytocin, social deficits

## Abstract

**Background:**

Negative symptoms are a core aspect of psychopathology in schizophrenia. Currently available pharmacological agents have proven minimally efficacious for remediating negative symptoms. A promising treatment avenue is the intranasal administration of the neuropeptide oxytocin. However, there have been inconsistencies in effects of oxytocin on negative symptoms throughout the literature, and factors leading to inconsistent effects are unclear.

**Methods:**

We conducted a systematic review and meta-analysis of randomized clinical trials to compare the effectiveness of oxytocin with placebo for the treatment of negative symptoms and determine moderators of treatment effect. Random effects meta-analyses and dose-response meta-analysis were performed on mean changes in negative symptoms.

**Results:**

In an initial analysis of all 9 identified randomized clinical trials, intranasal oxytocin showed no significant effect on negative symptoms. For higher doses (>40–80 IU), a beneficial effect on negative symptoms was found with a moderate effect size, but this effect disappeared after exclusion of 1 outlier study. The dose-response meta-analysis predicted that higher doses of oxytocin may be more efficacious for negative symptoms. For positive symptoms, no beneficial effect of oxytocin was found in the main meta-analysis, but the dose-response meta-analysis suggested a potential advantage of higher doses.

**Conclusions:**

The present results show no consistent beneficial effect of intranasal oxytocin for the treatment of negative and positive symptoms. The dose-response meta-analysis does not allow drawing any firm conclusions but suggests that high doses of intranasal oxytocin may be more efficacious. If future studies are conducted, an effort to reach adequate CNS concentrations for a sufficient duration is required.

**Significance Statement**

There is an urgent need for evidence-based interventions to improve negative symptoms, and intranasal oxytocin is a promising candidate. In our systematic review and meta-analysis, we did not find a benefit of oxytocin across all studies. However, both a subgroup meta-analysis and a dose-response meta-analysis showed that high doses of oxytocin of more than 40 IU/d could be more efficacious in the treatment of negative symptoms. Overall, the present meta-analysis suggests that at least 1 additional trial is needed that aims at reaching sufficient CNS concentrations using higher doses of oxytocin and potentially a more frequent administration as well as adherence monitoring.

## Introduction

The clinical manifestations of schizophrenia include positive, disorganized, and negative symptoms (Peralta et al., 1997). While positive symptoms can often be effectively managed with antipsychotic medications, negative symptoms are still an unmet clinical need. It has been proposed that negative symptoms cluster in 2 psychopathological dimensions: amotivation (anhedonia, avolition, asociality) and diminished expression (alogia, blunted affect) (Messinger et al., 2011; Guessoum et al., 2020). More recently, a 5-factor or hierarchical model has been shown to better reflect the structure of negative symptoms (Strauss et al., 2018). Negative symptoms are present in at least 50% of patients with schizophrenia and constitute a heavy burden, as they are associated with poor social and role functioning, reduced quality of life, and low rates of recovery (Rabinowitz et al., 2012; Aleman et al., 2017; Porcelli et al., 2019).

Therefore, the development of novel therapeutic approaches to alleviate these impairments is a mental health priority (Miyamoto et al., 2012). The neuropeptide oxytocin has shown prosocial effects and has therefore been proposed as a treatment for mental disorders characterized by social dysfunction, such as autism and schizophrenia (Meyer-Lindenberg et al., 2011; Kirsch, 2015). Preliminary results in healthy individuals have revealed that intranasal oxytocin administration facilitates behavioral and endocrine responses to social stress, attenuates amygdala reactivity to social stimuli and improves various aspects of social cognition (e.g., emotion recognition and empathy), and enhances social attachment (Meyer-Lindenberg et al., 2011; Riem et al., 2014).

Oxytocin has been investigated as a potential treatment for negative symptoms that are partially related to social dysfunction. Among negative symptoms, asociality is part of the amotivation dimension that emerges from an impaired motivation for social contact and may be defined as the lack of self-initiated social interactions (Messinger et al., 2011). Indeed, Strauss and colleagues found that the severity of asociality in patients with schizophrenia can be predicted by lower plasma oxytocin levels (Strauss et al., 2015). Furthermore, Haram and colleagues found a significant association between emotional withdrawal and 1 oxytocin receptor gene variant (Haram et al., 2015). These findings further support interest for oxytocin in the treatment of asociality as a core negative symptom of schizophrenia. In addition to negative symptoms, the positive symptom of persecutory delusions has also been considered a potential target for oxytocin treatment because of its strong social component (Brown et al., 2014; Yao et al., 2018).

Despite the anticipated potential of oxytocin for treating negative symptoms, the findings from individual studies have not been consistent, and 2 recent meta-analyses arrived at different but mostly negative conclusions (Oya et al., 2016; Williams and Burkner, 2017), with one of studies suggesting a potential effect for daily administration of oxytocin. The most recent meta-analysis also did not find an effect for negative symptoms but a reduction of positive symptoms with high doses of oxytocin (Zheng et al., 2019). However, the observed effect was mainly driven by a study including patients that were only partially stabilized (Modabbernia et al., 2013).

Thus, although the main findings of all meta-analyses are negative, 2 of them suggest a modulation depending on the frequency and dose of oxytocin administration. This concern regarding dose of treatment is of high potential relevance because high dosage of intra-nasal oxytocin has been shown to lead to increased cerebrospinal fluid concentrations in animals (Freeman et al., 2016). High doses up to 96 IU for a duration of 8 weeks have been shown to be safe in humans (Zhang et al., 2013). Despite this issue, to the best of our knowledge, no formal dose-response meta-analysis has yet been conducted.

We conducted a systematic review, a meta-analysis, and a dose-response meta-analysis of all randomized clinical trials (RCTs) investigating the effects of intranasal oxytocin as an add-on to antipsychotic treatment on the negative symptoms of schizophrenia. The primary hypothesis was that the add-on of intranasal oxytocin would lead to a reduction in negative symptoms compared with placebo add-on. We employed subgroup analyses and dose-response meta-analysis to investigate the impact of oxytocin dose. In addition, a secondary hypothesis was that add-on treatment would lead to a reduction in positive symptoms.

## Methods

### Registration

The current systematic review and meta-analysis followed the Preferred Reporting Items for Systematic Reviews and Meta-Analyses guidelines (Moher et al., 2009). The protocol included the rationale for the study, objectives, eligibility criteria, information sources, search strategy, data extraction, outcomes, methods for assessing study quality and risk of bias, strategy for data synthesis, and statistical methodology. On July 7, 2020, the protocol was published in the International Prospective Register of Systematic Reviews under registration number CRD42020160648.

### Search Strategy

A systematic search of published literature in the following databases was conducted: MEDLINE, PubMed, EMBASE, PsycINFO, PsycARTICLES, and Cochrane Database of Systematic Reviews. Trial registries (clinicaltrials.gov and clinicaltrialsregister.eu) were also searched for relevant articles. A combination of the following search terms was used: (oxytocin or oxt) and (schizo* or psychosis or psychotic). The following search limits were applied: English language and human studies. The conducted search covered publications until the end of July 2020. Previously published systematic reviews, meta-analyses, posters, and the reference lists of retrieved articles were closely examined for additional reports.

### Inclusion Criteria and Study Selection

**Figure 1.**
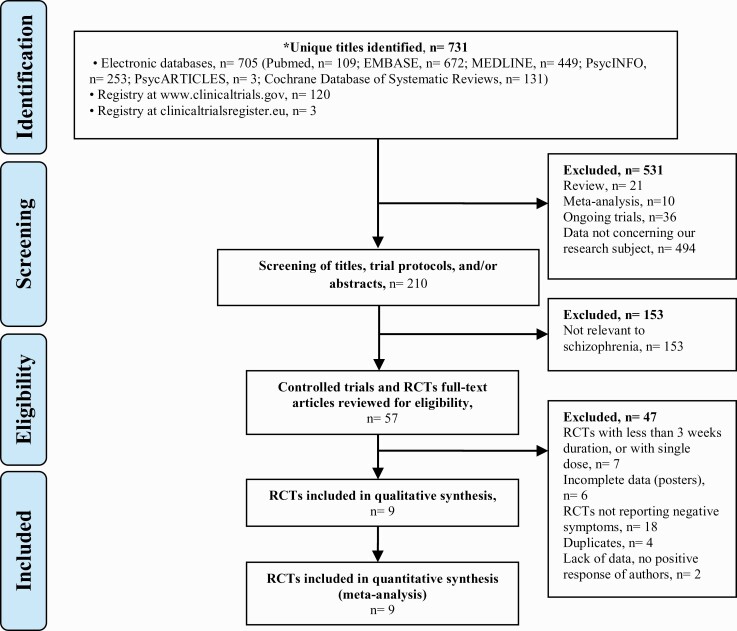
Systematic review PRISMA flow chart.

We included all double-blind RCTs in which oxytocin was compared with placebo. The intervention duration had to be at least 3 weeks, as the effects of treatments on negative symptoms need time to develop. Only articles reporting intranasal oxytocin were included. Pilot dose studies, open-label clinical trials, case series, and reports were excluded (Figure 1). For identified crossover studies, a detailed examination of methodology was conducted to assess possible risks of carry-over effects on the primary outcome. Two of the authors (M.S. and N.Z.) independently examined titles and abstracts of all papers identified in the electronic searches that were possibly appropriate before assessing the full text and references of all shortlisted articles.

### Outcomes and Data Extraction

The primary outcome was the mean change in negative symptom scores. All available scales for assessing negative symptoms were considered, such as the negative subscale of the Positive and Negative Syndrome Scale (PANSS-N) (Kay et al., 1987), the Scale for Assessment of Negative Symptoms (SANS) (Andreasen, 1989), the Comprehensive Assessment Interview for Negative Symptoms (CAINS) (Kring et al., 2013), the withdrawal/retardation factor of the Brief Psychiatric Rating Scale (BPRS) (Overall and Gorham, 1962), the Brief Negative Symptom Scale (BNSS) (Kirkpatrick et al., 2011), and the Negative Symptom Assessment (Alphs et al., 1989).

The secondary outcome was the mean change in positive symptom scores. The retained scales were the positive subscale of the PANSS, the Scale for the Assessment of Positive Symptoms (Andreasen, 1984), and the sum of the thinking/disturbance and hostile/suspiciousness factors of the BPRS. To allow comparison between studies, all scores were converted to PANSS scores (van Erp et al., 2014).

Two authors (M.S. and N.Z.) independently conducted the data extraction, and any discrepancies were resolved with a common full-text review with the third author (S.K.). Different variables were extracted from the selected studies: (1) paper information (author, year), (2) design of the trial, (3) population, (4) trial procedure, (5) type of treatment and control, and (6) bias assessment based on reading the full text. The Cochrane Collaboration Risk of Bias Assessment Tool was used to assess the methodological quality of the included studies. It included sequence generation, allocation concealment, blinding of participants and personnel, blinding of outcome assessment, incomplete outcome data, selective reporting, and other potential sources of bias.

### Statistical Analysis

The Cochrane Collaboration software Review Manager (RevMan, version 5.3) was used for our meta-analysis. The effect sizes were calculated as standardized mean differences (SMDs) because heterogeneous samples of scales were employed in the included studies for all considered outcomes. Three studies reported mean changes (Modabbernia et al., 2013; Buchanan et al., 2017; Jarskog et al., 2017), and 2 of these studies reported baseline, endpoint, and mean changes for our primary outcome (Modabbernia et al., 2013; Buchanan et al., 2017). Hence, we were able to impute all mean changes by applying the correlation matrix extracted from Buchanan et al. (2017) by applying the method described by Abrams and colleagues (Abrams et al., 2005). We chose to use only the correlation matrix of this study because Modabbernia et al. (Modabbernia et al., 2013) included patients who were only partially stabilized. Additionally, regarding the positive symptoms, the mean change was extracted with the use of 1 study. Nonetheless, values concerning positive symptoms were not available for 2 studies (Davis et al., 2014; Buchanan et al., 2017). Furthermore, since different doses were used and the trial durations and selected doses were quite heterogeneous among studies, a random effects model was applied. We conducted an exploratory analysis considering subgroups of treatment doses since 24- to 40-IU doses were used. The I^2^ statistic was used to measure the inconsistency across studies’ results. In addition, we conducted a dose-response meta-analysis by following the 1-stage approach proposed by Crippa and Orsini (Crippa et al., 2019). This method estimates a combined dose-response association considering the correlation among a set of mean differences. The pooled curve and estimates of the between-studies heterogeneity are based on the whole set of studies.

We characterized the dose-response relation using a restricted cubic spline model with 3 knots located at the 10th, 50th, and 90th percentiles of the overall dose distribution. These analyses were carried out using R software version 3.1 with the metafor (Viechtbauer, 2010) and dosresmeta packages (Crippa and Orsini, 2016).

## Results

### Search Results

The systematic search yielded 731 unique references. In accordance with our protocol, 531 titles were excluded, and 210 clinical trial titles, protocols, and abstracts were screened. Thus, we conducted a full-text review for eligibility for 57 controlled trials. Finally, 9 RCTs were included in our qualitative analysis. Seven studies had to be excluded for insufficient trial and dose duration. Six posters were found, of which 2 referred to unpublished data, raising concerns of potential publication bias.

### Qualitative Description of Included Studies

In sum, 9 RCTs using intranasal oxytocin met our inclusion criteria. No RCTs using other modes of administration were found. All 9 trials on intranasal oxytocin could be included in the meta-analysis of our primary outcome regarding the mean change in negative symptoms. However, for positive symptoms as our secondary outcome, 3 studies could not be included because the information on positive symptoms was incomplete (Davis et al., 2014; Cacciotti-Saija et al., 2015; Buchanan et al., 2017). Of importance, in 1 study, participants were instructed to self-administer the treatment only before each social cognitive training session (Davis et al., 2014), but we decided to keep this study in the main analyses in line with previous meta-analyses and to conduct a sensitivity analysis without this study.

The included patients were consistently between 18 and 65 years of age, albeit 1 study included patients from 16 to 35 years (Cacciotti-Saija et al., 2015). Among all studies, the mean average age was 27.1 years. The included patients had been diagnosed with schizophrenia, schizoaffective, or schizophreniform disorder (DSM IV, IV-TR); the mean duration of illness was 12.6 years. One study had a sample consisting of only male patients (Davis et al., 2014), and all other studies had a majority of men (72.6%). The mean duration of the intervention was 7.3 weeks, ranging from 3 weeks (Feifel et al., 2010) to 16 weeks (Jarskog et al., 2017).

The mean dose of intranasal oxytocin was 47.6 IU, ranging from 24 IU to 80 IU (Table 1). Since intranasal oxytocin was self-administered and a majority of the participants were outpatients (minimum, 53%), 3 studies recorded compliance with treatment (Gibson et al., 2014; Cacciotti-Saija et al., 2015; Jarskog et al., 2017), leading to an estimated 79.7% treatment compliance. One study required a minimum of 75% compliance assessed by weekly dose count and weight of returned intranasal bottles to avoid exclusion from the trial (Buchanan et al., 2017).

**Table 1.** Characteristic of Included RCTs

Study (author, year)	Type of study	Inclusion criteria	Scale used for assessment of positive and negative symptoms	Intervention and control condition	Groups mean	N (male)	Mean age, y	Mean duration of illness	Estimated compliance to treatment
20 IU/ twice per day									
Lee et al. 2013 (US)	3 wk, double-blind, placebo controlled	Inpatients and outpatients with schizophrenia or schizoaffective disorder (DSM-IV). On stable medication(s) and dose(s) for at least 6 wk. Exclusion of patients affected by substance abuse disorders in last month. 18–60 y	SANS, BPRS	Self-administration of 20 IU oxytocin or placebo intra-nasally twice daily. Each 20-IU oxytocin dose consisted of 5 puffs, each containing 4 IU oxytocin.	Oxytocin group Placebo group	13 (75%) 15 (83.3%)	44.74 ± 11.74 35.07 ± 8.21	n.a. n.a.	n.a. n.a.
24 IU/ twice per day									
Cacciotti et al. 2015 (Australia)	6 wk, double-blind, placebo controlled	Outpatients with diagnosis of schizophrenia, schizophreniform disorder, or schizoaffective disorder within the first 3 years of illness onset. Exclusion of patients with substance abuse disorders. Medication was stable for at least 8 wk prior to entering study. 16–35 y	SANS	Self-administration of intranasal oxytocin (24 IU) or placebo twice-daily. Participants instructed to administer 2 sprays (1 per nostril) morning and night, with each spray containing 12 IU. Additional 24 IU was administered 15 min prior to each weekly social cognition training session. Both groups received social cognition training (total of 12 h).	Oxytocin group Placebo group	27 (75%) 25 (83.3%)	21.52 ± 4.22 22.32 ± 4.43	16.0 ± 11.8 12.89 ± 9.43	92.6% 84.0%
24 IU/ twice per day									
Gibson et al. 2014 (US)	6 wk, double-blind, placebo controlled	Inpatients and outpatients with diagnosis of schizophrenia (DSM-IV-TR); stability of symptom severity; at least moderate clinical psychiatric symptoms as defined by total PANSS score >60; social difficulty defined by PANSS score ≥4 on suspiciousness/paranoia item, or a 3 on suspiciousness/paranoia item and 3 or higher on 1 of the other socially relevant PANSS items; low to moderate depressive symptoms; on same medication(s) and dose(s) for at least 1 month prior to study participation. 18–55 y	PANSS	Self-administered intranasal study drug twice daily (before breakfast and before dinner). Each dose consisted of six 0.1-mL insufflations (alternating every 30 sec between left and right nostril) of OT spray; total insufflation at each dose approximately 24 IU of OT (Syntocinon spray) or placebo.	Oxytocin group Placebo group	8 (69%) 6 (73%)	38.8 ± 7.22 35.67 ± 9.00	16.0 ± 11.8 12.89 ± 9.43	86.6% 76.5%
Jarskog et al. 2017 (US)	12 wk, double-blind, placebo controlled	Outpatients with schizophrenia or schizoaffective disorder (DSM-IV). Patients clinically stable for 1 month, with minimum duration of illness of 1 y. Patients had baseline deficit in social cognition (<24 on Reading the Mind in the Eyes Test) or in symptoms relating to social functioning (≥3 on 2 or more of following PANSS items: suspiciousness/persecution and hostility, passive/apathetic social withdrawal, uncooperativeness, active social avoidance). Exclusion of patients with substance abuse disorders during last 3 mo. 18–65 y	PANSS	Self-administered intranasal study drug twice daily (before breakfast and before dinner). Each dose consisted of six 0.1 mL insufflations (alternating every 30 seconds between the left and right nostril); each dose was approximately 24 IU of oxytocin (Syntocinon spray) or placebo.	Oxytocin group Placebo group	32 (75%) 30 (76.7%)	n.a.	16.0 ± 11.8 12.89 ± 9.43	75.0% 81.2%
Buchanan et al. 2017 (US)	6 wk, double-blind, placebo controlled	Inpatients and outpatients with schizophrenia or schizoaffective disorder (DSM-IV-TR). Clinically stable patients with persistent negative symptoms based on 4-wk evaluation phase with: (SANS) total score ≥20 or alogia global score ≥3, BPRS positive symptom total score ≤16); affective symptoms (BPRS Anxiety/Depression factor score ≤14); and extrapyramidal symptoms (SAS total score ≤10). Stable treatment for 2 mo and with no substance use disorder criteria in past 6 mo. 18–65 y	SANS, BPRS	Self-administered intranasal study drug twice daily (24 IU) (syntocinon spray) and matching intranasal placebo oxytocin.	Oxytocin group Placebo group	16 (87.5%) 20 (85.0%)	47.4 ± 11.2 42.2 ± 11.7	25.7 ± n.a. 23 ± n.a.	>75.0% >75.0%
40 IU/ once daily									
Dagani et al. 2016 (US)	8 mo, double-blind, placebo-controlled cross-over	Inpatients and outpatients with a diagnosis of schizophrenia (DSM-IV). PANSS score at least 55 and a (CGI-S) scale score at least 4 (moderately ill) at randomization required for inclusion. No substance use disorder criteria in past year. 18–45 y	PANSS	Self-administered 4 months of daily intranasal oxytocin (syntocinon spray) and 4 mo of daily intranasal placebo, with 1 wk of washout in between. Oxytocin was dosed at 40 IU (10 sprays) once a day. A caregiver monitored administrations.	Oxytocin group Placebo group	8 (81.3%)	30.4 ± 6.7	7.8 ± 2.6	n.a. n.a.
20–40 IU/ twice per week									
Davis et al. 2014 (US)	6 wk, double-blind, placebo controlled	Outpatients with schizophrenia (DSM-IV). Patients clinically stable on an antipsychotic medication with no dose change within 3 mo of study entry. 18–65 y	CAINS, BPRS	Nasal sprays were prepared in 30-mL multi-use bottles, calibrated to dispense 0.1 mL per puff. Subjects were instructed to spray 4 puffs into each nostril, for a total dose of 40 IU or equivalent placebo before each social cognitive training (total of 12 h).	Oxytocin group Placebo group	13 (100%) 14 (100%)	37.0 ± 10.8 42.8 ± 9.1	25.7 ± n.a. 23 ± n.a.	>75.0% >75.0%
Feifel et al. 2010 (US)	3 wk, double-blind, placebo-controlled cross-over design	Patients with diagnosis of schizophrenia (DSM-IV). Stable medications in previous 4 wk. PANSS score of at least 55 and CGI-S scale score of at least 4 (moderately ill). Score of at least 4 (moderate) on item 6 suspiciousness/persecution of PANSS. ≥18 y	PANSS	Subjects received 3 weeks of daily intranasal oxytocin (syntocinon spray) and 3 wk of daily intranasal placebo. Oxytocin was dosed at 20 IU (5 sprays) twice a day for the first week and 40 IU (10 sprays) twice a day thereafter.	Oxytocin group Placebo group	16 (87.5%) 20 (85.0%)	47.4 ± 11.2 42.2 ± 11.7	25.7 ± n.a. 23 ± n.a.	n.a. n.a.
Modabbernia et al. 2013 (Iran)	8 wk, double-blind, placebo controlled	Inpatients with schizophrenia or schizoaffective disorder (DSM-IV-TR). Patients required to be treated with stable dose of risperidone for at least 4 wk and had been partially stabilized (<20% change on PANSS in 2 subsequent visits 1 wk apart) and have significant residual symptoms as defined by minimum PANSS score of 60. No substance use disorder criteria in past 6 mo. 18–50 y	PANSS	Oxytocin spray was administered as 20 IU (5 syntocinon sprays) twice a day for the first week followed by 40 IU (10 sprays) twice a day for the following weeks.	Oxytocin group Placebo group	20 (85.0%) 20 (80.0%)	32.3 ± 7.4 33.2 ± 6.9	6.2 ± 5.1 6.5 ± 5.3	n.a. n.a.

Abbreviations: BPRS, Brief Psychiatric Rating Scale; CGI-S, Clinical Global Impression scale-severity; n.a., not available; PANSS, Positive and Negative Syndrome Scale; SAS, Simpson-Angus Extrapyramidal Symptom Rating Scale ; SANS, Scale for the Assessment of Negative Symptoms.

Furthermore, 3 studies included clinically stabilized patients for a mean period of 6 weeks (Davis et al., 2014; Buchanan et al., 2017; Jarskog et al., 2017), and 1 study included “partially” stabilized patients (<20% change in the PANSS, in 2 subsequent visits 1 week apart, after 4 weeks with the same antipsychotic medication) (Modabbernia et al., 2013). Six studies included patients with a stable medication over a mean period of 6.25 weeks. Only 1 study failed to define clinical stability or medication stability at inclusion (Dagani et al., 2016). Moreover, 1 study included patients with predominant negative symptoms (Buchanan et al., 2017). Three studies included patients presenting a specific PANSS total score, that is, a minimum score of 55 (Feifel et al., 2010) or a score ≥3 on 2 or more of the following items: suspiciousness/persecution, hostility, passive/apathetic social withdrawal, uncooperativeness, and active social avoidance (Gibson et al., 2014; Jarskog et al., 2017).

Finally, our bias assessment with the Cochrane collaboration risk of bias tool revealed that most studies had a low risk of bias (supplementary Table 1).

### Intranasal Oxytocin Effects on Negative Symptoms

**Figure 2.**
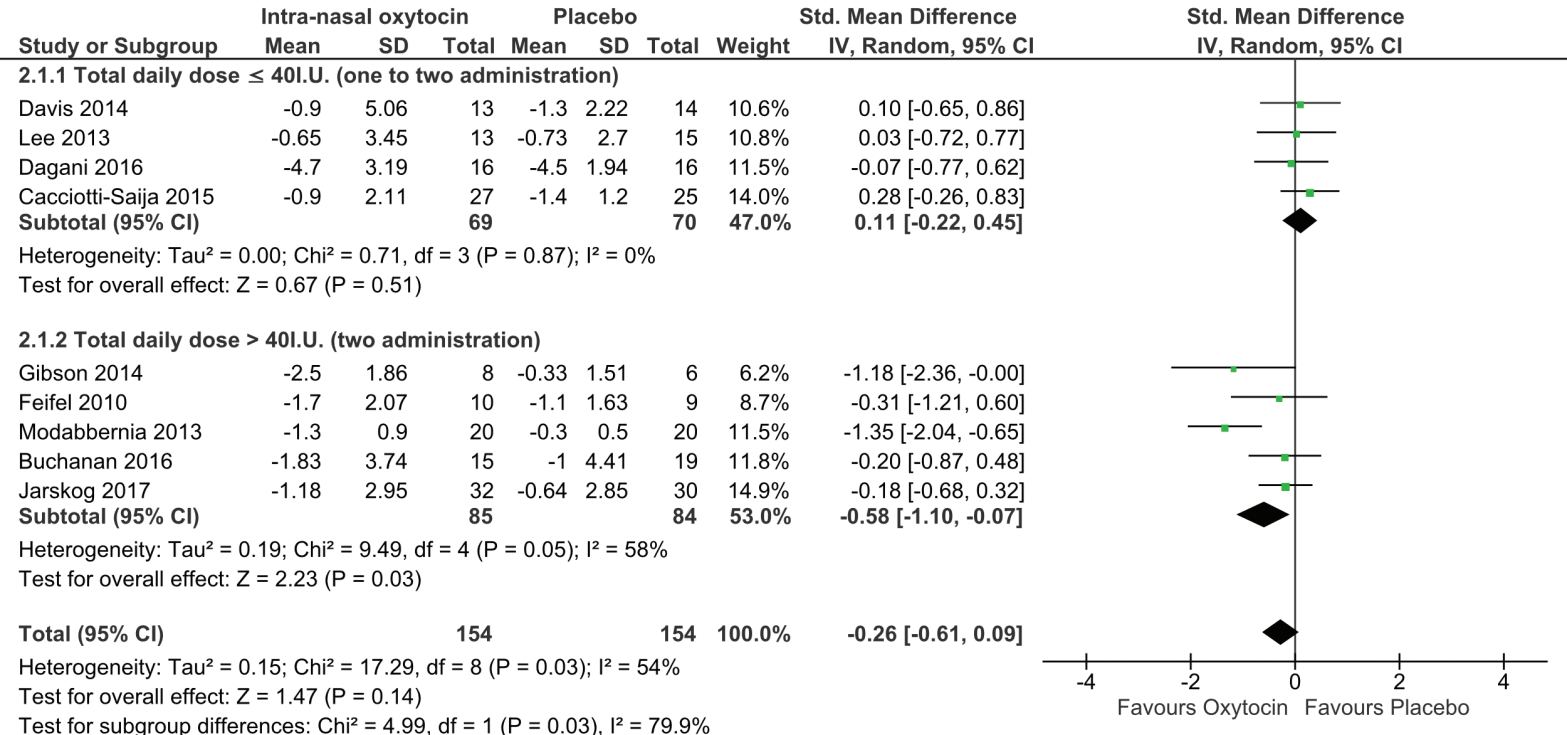
Forest plot for negative symptoms including the subgroup analysis according to daily dose of oxytocin.

Our primary outcome concerning negative symptoms was reported for all retained studies with different scales (PANSS, SANS, CAINS). Our main results revealed that intranasal oxytocin was not superior to placebo with respect to the mean change in negative symptoms (9 RCTs; n = 308; SMD = −0.26; 95% CI = −0.61 to +0.09; *P* = .14). The effect size was small, and moderate heterogeneity was present (I^2^ = 54%) (Figure 2). The corresponding funnel plot was not in favor of asymmetry but suggested 2 outliers (Modabbernia et al., 2013; Gibson et al., 2014) (supplementary Figure 1). A sensitivity analysis revealed that the exclusion of Modabbernia et al. (2013) lowered heterogeneity (I^2^ = 8%) and the effect size (SMD = −0.08; 95% CI = −0.32 to +0.16; *P* = .52). This study included partially stabilized patients and was the only study conducted in Iran. In addition, the exclusion of the Davis et al. (2014) study, where participants only administered oxytocin prior to social cognitive training, did not change the results (SMD = −0.31; 95% CI = −0.69 to +0.07; *P* = .11).

Furthermore, 2 studies reported scores for the amotivation dimension using the SANS scale (Lee et al., 2013) and the CAINS (Davis et al., 2014). The effect was small and nonsignificant (2 RCTs; n = 56; SMD = −0.11; 95% CI = −0.64 to +0.41; *P* = .64) (supplementary Figure 2). Only 1 study reported specific effects for asociality, but the effects were small and non-significant (1 RCT; n = 28, SMD= −0.23; 95% CI = −2.77 to 2.32; *P* = .86) (Lee et al., 2013).

### Secondary Outcome: Effects of Intranasal Oxytocin on Positive Symptoms

Our secondary outcome focusing on the mean change in positive symptoms was extracted for 7 of the retained studies. Different scales were used to report this outcome (positive subscale of the PANSS, Scale for the Assessment of Positive Symptoms). An initial overall analysis showed no significant effect of intranasal oxytocin on positive symptoms (SMD = −0.04; 95% CI = −0.46 to +0.38; *P* = .85) in the presence of moderate heterogeneity (I^2^ = 60%).

### Subgroup Analysis for the Effects of Oxytocin Dose on Negative Symptoms

Since the retained studies were heterogeneous with respect to oxytocin doses, a subgroup analysis focusing on daily total dose was conducted. Two subgroups were constituted: one with studies employing a mean total dose ≤40 IU/d divided into 1 to 2 administrations of intranasal oxytocin (low-dose group), and the second one with a higher mean total dose per day divided into 2 administrations (>40 IU) (high-dose group) (Figure 2). The results revealed nonsignificant results for the low-dose subgroup (SMD = 0.11; 95% CI = −0.22 to +0.45; *P* = .51) in the absence of heterogeneity (I^2^ = 0%). The exclusion of the Davis et al. (2014) study did not change the results for the low-dose subgroup (SMD = +0.12; 95% CI = −0.26 to +0.49; *P* = .54).

The analysis for the high-dose group suggested a beneficial effect of intranasal oxytocin on the mean change in negative symptoms with a moderate effect size (SMD = −0.58; 95% CI = −1.10 to −0.07; *P* = .03) in the presence of moderate heterogeneity (I^2^ = 58%). However, the exclusion of the Modabbernia et al. (2013) study as a potential outlier led to trend-level results with a small effect size (SMD = −0.29; 95% CI = −0.64 to +0.06; *P* = .10) and made the within-subgroup heterogeneity disappear (I^2^ = 0%).

### Subgroup Analysis for the Effects of Oxytocin Dose on Positive Symptoms

**Figure 3.**
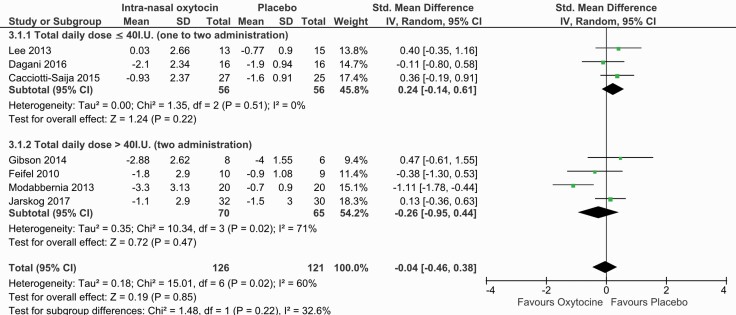
Forest plot for positive symptoms including the subgroup analysis according to daily dose of oxytocin.

As we did for our primary outcome, we explored heterogeneity with a subgroup analysis based on oxytocin dose (Figure 3). For the low-dose group (≤40 IU), the results were nonsignificant (SMD = 0.24; 95% CI = −0.14 to +0.61; *P* = .51) in the absence of within-group heterogeneity (I^2^ = 0%). Similarly, for the high-dose subgroup (>40 IU), the results were nonsignificant (SMD = −0.26; 95% CI = −0.95 to +0.44; *P* = .47) in the presence of moderate within-group heterogeneity (I^2 ^=^ ^71%). The Modabbernia et al. (2013) study appeared as an outlier in the corresponding funnel plot (supplementary Figure 3). The exclusion of this study eliminated overall heterogeneity (I^2 = ^0%) without changing the results (SMD = 0.16; 95% CI = −0.11 to +0.44; *P* = .24).

### Dose-Response Meta-Analysis for Negative Symptoms

To further explore the dose-dependency relationship of intranasal oxytocin with negative symptoms, we conducted a dose-response meta-analysis. We used a non-linear model that pooled dose-response associations between fixed doses of oxytocin (40, 48, 80 IU/d) and the mean change in the negative subscale scores of the PANSS in the 9 included studies.

**Figure 4.**
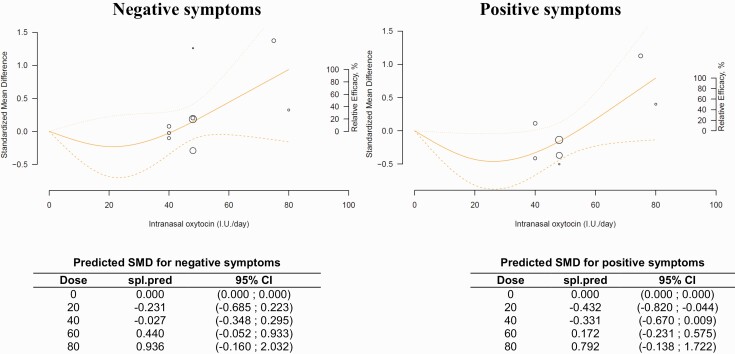
Dose-response meta-analysis for the relationship between oxytocin dose, negative symptoms (n = 9), and positive symptoms (n = 7). The figure represents a pooled dose-response association between doses of oxytocin in IU/day and the mean change in the negative subscale scores of the PANSS (solid line). The oxytocin dosage is modeled with restricted cubic splines in a random-effects model. Dashed lines represent the 95% confidence intervals for the spline model. Circles indicate observed mean differences in individual studies; size of bubbles is proportional to precision (inverse of variance) of the standardized mean differences. Right axis represents percentage of the maximum predicted effect. Predicted standardized mean difference for negative and positive symptoms are reported below the figure in a table.

The model-based prediction suggests that doses of intranasal oxytocin ≤40 IU did not reduce negative symptoms, with an inversed bell-shaped curve (Figure 4). However, the model suggests that efficacy increases from 40 to 80 IU (40 IU: −0.027 [95% CI = −0.348 to +0.295]; 80 IU: +0.936 [95% CI = −0.16 to +2.03]) (Figure 4). It should be noted that the curve was still ascending at doses of 80 IU, suggesting that higher doses could be more efficacious for negative symptoms. The estimated doses to produce 50% and 95% of the predicted maximum effect were ED50 = 61.1 IU and ED95 = 78.1 IU. The pooled predicted dose-response curve and the confidence intervals and the model mean differences are provided in Figure 4.

To inspect the reliability of our results, we conducted a sensitivity analysis with the exclusion of the Modabbernia study (2013). For negative symptoms, the exclusion of this study did not lead to a major change in the dose-response curve. Slight decreases of the ED50 to 58 IU and ED95 to 77.8 IU were found, suggesting only a minor impact on results (supplementary Figure 4).

### Dose-Response Meta-Analysis for Positive Symptoms

For positive symptoms, 7 studies were available. The model-based predicted that doses <60 IU intranasal oxytocin were not efficacious for positive symptoms, with an inversed bell-shaped curve (Figure 4). For doses of 60 to 80 IU, the model suggested increasing effects on positive symptoms (60 IU: +0.172 [95% CI = −0.231 to +0.575]; 80 IU: +0.792 [95% CI = −0.138 to +1.722]). For higher doses, the curve was still ascending, suggesting that higher doses could be more efficacious for positive symptoms (Figure 4). The estimated doses to produce 50% and 95% of the predicted maximum effect were higher than for negative symptoms, with the ED50 = 67.2 IU and the ED95 = 78.7 IU The sensitivity analysis excluding the Modabbernia study (2013) had only a limited impact on the dose-response curve with a small increase of ED50 and ED95 to 70.1 and 79 IU, respectively (supplementary Material; Figure 4).

## Discussion

### Effects of Intranasal Oxytocin on Negative Symptoms

In the present meta-analysis, we did not find a beneficial overall effect of intranasal oxytocin on negative symptoms. A significant reduction in negative symptoms was found for the high-dose subgroup (>40 IU) (Figure 2), which is consistent with the findings of the dose-response meta-analysis suggesting that the efficacy of intranasal oxytocin increases with high doses of oxytocin. However, for the subgroup analysis, our results with higher doses of intranasal oxytocin for negative symptoms were mainly driven by 1 study that included partially stabilized patients (Modabbernia et al., 2013). This study was identified as a potential outlier in the funnel plot and had an unusually high effect size. Exclusion of this study led to nonsignificant results for the subgroup analysis but did not have a major impact on the results of the dose-response meta-analysis.

In contrast to the (Williams and Buckner, 2017) as well as the (Zheng et al. 2019) meta-analyses that reported the absence of efficacy of intranasal oxytocin on negative symptoms, our meta-analysis suggests a possible trend-level effect on negative symptoms of high-dose oxytocin, even after the exclusion of the Modabbernia study (2013). Noteworthy, 1 main relevant difference from previous meta-analyses was our decision to select mean changes as an outcome parameter, which is more robust to baseline differences in small studies. In addition, compared with the latest meta-analysis conducted on the subject (Zheng et al., 2019), we decided to include only Gibson et al. (2014) and not Pederson et al. ( 2011), which report findings from the same trial with an overlap of inclusions.

It is important that most of the included studies did not specifically enroll patients with predominant or persistent negative symptoms. Only (Buchanan et al. 2017) specifically included patients with predominant negative symptoms, and this is also most in line with recommendations for treatment trials targeting negative symptoms. The authors found a small and nonsignificant effect of oxytocin compared with placebo. However, no conclusions about the dose-response association can be drawn for predominant or persistent negative symptoms.

### Effects of Intranasal Oxytocin on Positive Symptoms

Although 2 studies in our sample did not report scores regarding our secondary outcome, the results obtained concerning the effect of intranasal oxytocin on positive symptoms clearly showed that intranasal oxytocin had no benefit on positive symptoms. Our results diverge from the Zheng and colleagues meta-analysis (Zheng et al., 2019) regarding positive symptoms, which suggests that high doses are efficacious on positive symptoms reduction. However, their results must be interpreted with caution as no sensitivity analysis—with exclusion of the Modabbernia et al. (2013) study—was conducted, and both Gibson et al. (2014) and Pederson et al. (2011) were included as stated above. While the results of our subgroup analysis diverge from Zheng et al. (2019), our dose-response model predicts that higher doses of oxytocin could be more efficacious on positive symptoms even with exclusion of the outlier study. Although our dose-related findings are less clear for positive than for negative symptoms, it is possible that higher doses could have a beneficial effect.

### The Potential Role of Oxytocin Dose and Frequency of Administration

The levels of CNS oxytocin reached after intranasal oxytocin application remain a matter of debate (Quintana et al., 2020). In this context, the roles of oxytocin dosage and frequency of administration are of high interest. Our negative results for standard doses of oxytocin might appear to be in contrast to single-dose administration or short-term studies reporting improvement in symptoms of schizophrenia (Goldman et al., 2011; Davis et al., 2013; Horta de Macedo et al., 2014). While our subgroup analysis suggests an advantage for higher doses of oxytocin only for negative symptoms, the dose-response meta-analysis suggests that higher doses could be more efficacious on both negative and positive symptoms. However, to obtain a clinical effect, a more frequent administration of intranasal oxytocin may be necessary in addition to a higher dose.

Indeed, 1 critical issue in this respect concerns the pharmacokinetic profile of intranasal oxytocin. The half-life of oxytocin is approximately 2 hours. One study showed that administration of 24 IU resulted in elevated plasma oxytocin levels from 15 to 75 minutes and elevated cerebrospinal fluid levels at 75 minutes (Striepens et al., 2013). Therefore, considering that administration was limited to a maximum of 2 doses per day and doses were often small in the included studies, it is questionable whether CNS levels were sufficient to produce clinical effects on negative symptoms. In addition to dose and frequency, the device used for administration may have to be optimized in order to improve nasal deposition in the target regions essential for nose-to-brain transport (Djupesland and Skretting, 2012).

Discussions around optimal doses of oxytocin for schizophrenia are currently ongoing (Wynn et al., 2019). In this context, compliance with oxytocin treatment is also a critical issue and was adequately monitored in only some studies. Furthermore, the duration of treatment may be an important factor, because the effects of chronic oxytocin administration have been suggested to differ strongly from those of acute administration (Bradley and Woolley, 2017). Aside from these factors relating to treatment, individual factors may play an important role for the response to oxytocin response, such as gene expression, sex, age, antipsychotic treatment, early life events, and attachment style (Souza et al., 2010; Teltsh et al., 2012; Bradley and Woolley, 2017).

### Does Intranasal Oxytocin Target Specific Negative Symptoms?

Another important question regards the fact that oxytocin might not target global negative symptoms but has a rather specific effect on asociality (Lee et al., 2013). Only 1 of the included studies specifically reported individual symptom scores for asociality, revealing a small improvement only for inpatients (Lee et al., 2013). Only 2 studies reported amotivation factor scores (Lee et al., 2013; Davis et al., 2014) with no apparent effect in the aggregated results. For this purpose, rating scales allowing the assessment of the 5 negative symptoms as well as the 2 dimensions of amotivation and diminished expression should be employed in future studies (Kirkpatrick et al., 2011; Kring et al., 2013).

It has been suggested that oxytocin should be combined with social cognitive skills training. In our sample of studies, 2 studies found no advantage for oxytocin over placebo when combining this drug treatment with social cognitive skills training, but the doses of oxytocin were relatively low (40–48 IU/d), the duration was limited, and the enrollment criteria did not require patients to have primary and enduring negative symptoms (Davis et al., 2014; Cacciotti-Saija et al., 2015).

### Further Limitations

In addition to the critical issues outlined in the preceding section, the most evident limitation of our meta-analysis was the limited number of studies and participants included (n = 308). Differences in doses, administration of treatment, duration of trials, inclusion criteria, and selected outcomes contributed to the heterogeneity of our sample of studies. One addition may be that most included studies did not define negative symptoms as the primary outcome and the levels of negative symptoms at intake were limited. Recent meta-analyses on psychological and pharmacological treatments suggest that effects on negative symptoms might be stronger when those are the primary outcome and/or patients are selected based on the severity of negative symptoms (Sabe et al., 2019; Riehle et al., 2020). Importantly, in our sample, only 1 study selected participants with predominant negative symptoms after screening participants for secondary negative symptoms as recommended, but it had a short trial duration of 6 weeks. Nonetheless, the effects on negative symptoms need time to develop, and a 12- to 26-month duration has been recommended for clinical trials (Marder et al., 2020).

## Conclusions

The present results for the whole sample of RCTs suggest that short-term use of intranasal oxytocin is not effective for reducing the negative or positive symptoms of schizophrenia. However, our subgroup analysis for high doses of oxytocin (>40 IU) yielded a trend-level result with a small effect on negative symptoms. This result is also present in our dose-response meta-analysis that suggests that higher dose could be more efficacious. In view of the pharmacokinetic profile of intranasal oxytocin and our results, an effort to reach adequate CNS concentrations for a sufficient duration is required, including optimal administration frequency and compliance monitoring. We suggest that at least 1 more RCT with a high dose of intranasal oxytocin and more frequent administration should be carried out before firm conclusions can be drawn about the effect of intranasal oxytocin on negative symptoms. Importantly, the trial should include patients with predominant or persistent negative symptoms, and all individual negative symptoms—in particular asociality—should be assessed.

## Supplementary Material

pyab020_suppl_Supplementary_Figure_S1

pyab020_suppl_Supplementary_Figure_S2

pyab020_suppl_Supplementary_Figure_S3

pyab020_suppl_Supplementary_Figure_S4

pyab020_suppl_Supplementary_Table_S1
