# Supplementary material for: Intranasal Oxytocin for Negative Symptoms of Schizophrenia: Systematic Review, Meta-Analysis, and Dose-Response Meta-Analysis of Randomized Controlled Trials
Source: Int J Neuropsychopharmacol. 2021 Apr 23;24(8):601–14. doi: 10.1093/ijnp/pyab020 (PMC8378078; doi:10.1093/ijnp/pyab020)
Supplement: pyab020_suppl_Supplementary_Figure_S1 [file pyab020_suppl_supplementary_figure_s1.docx]

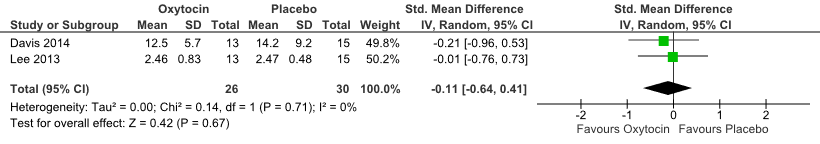


**Supplementary Figure S1.** Forest plot for studies for the effects of intra-nasal oxytocin on the amotivation dimensions of negative symptoms. Effect sizes are mean standardized differences at endpoint between intranasal oxytocin and placebo groups.
