# Supplementary figures and images for: Intranasal Oxytocin for Negative Symptoms of Schizophrenia: Systematic Review, Meta-Analysis, and Dose-Response Meta-Analysis of Randomized Controlled Trials
Source: Int J Neuropsychopharmacol. 2021 Apr 23;24(8):601–14. doi: 10.1093/ijnp/pyab020 (PMC8378078; doi:10.1093/ijnp/pyab020)

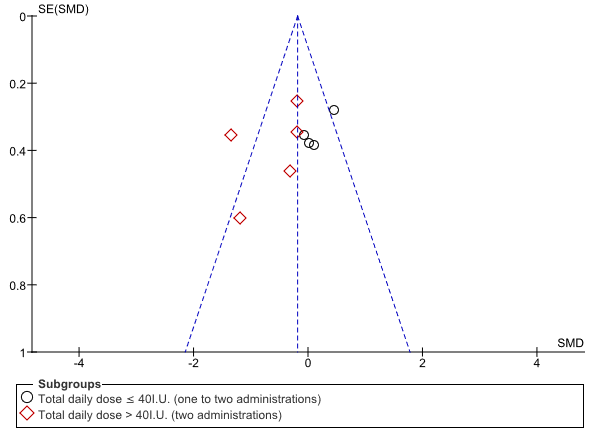


**Supplementary Figure S2.** Funnel plot for negative symptoms

Supplement: pyab020_suppl_Supplementary_Figure_S2 [file pyab020_suppl_supplementary_figure_s2.docx]

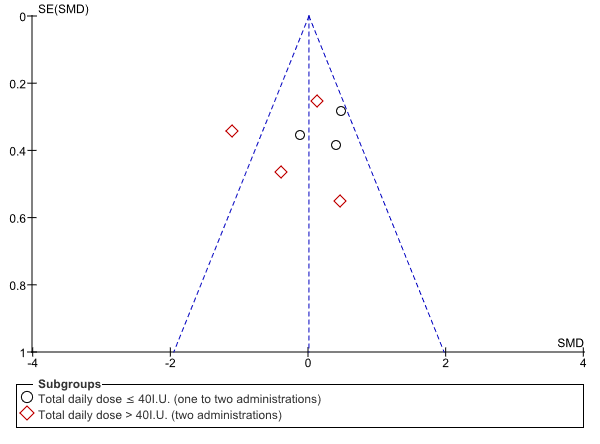


**Supplementary Figure S3.** Funnel plot for positive symptoms

Supplement: pyab020_suppl_Supplementary_Figure_S3 [file pyab020_suppl_supplementary_figure_s3.docx]
