# Supplementary material for: Intranasal Oxytocin for Negative Symptoms of Schizophrenia: Systematic Review, Meta-Analysis, and Dose-Response Meta-Analysis of Randomized Controlled Trials
Source: Int J Neuropsychopharmacol. 2021 Apr 23;24(8):601–14. doi: 10.1093/ijnp/pyab020 (PMC8378078; doi:10.1093/ijnp/pyab020)
Supplement: pyab020_suppl_Supplementary_Figure_S4 [file pyab020_suppl_supplementary_figure_s4.docx]

| **Negative symptoms** | **Positive symptoms** |
| --- | --- |
| 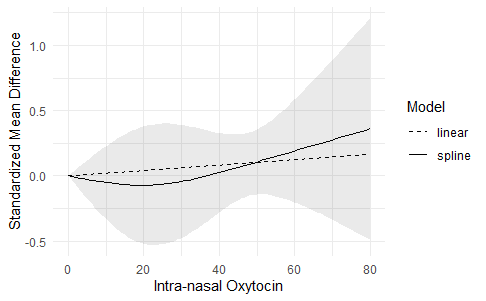 | 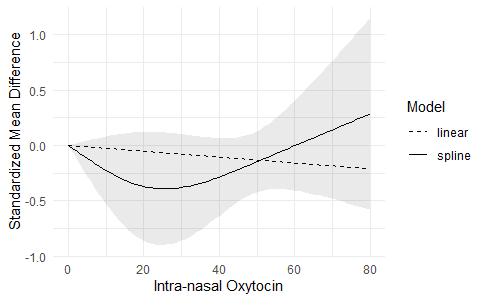 |

| **Predicted SMD for negative symptoms** | | |  | **Predicted SMD for positive symptoms** | | |
| --- | --- | --- | --- | --- | --- | --- |
| **Dose** | **spl.pred** | **95% CI** |  | **Dose** | **spl.pred** | **95% CI** |
| 0 | 0.000 | (0.000 ; 0.000) |  | 0 | 0.000 | (0.000 ; 0.000) |
| 20 | -0.072 | (-0.520; 0.376) |  | 20 | -0.432 | (-0.862 ; 0.120) |
| 40 | 0.023 | (-0.282; 0.328) |  | 40 | -0.331 | (-0.638 ; 0.064) |
| 60 | 0.190 | (-0.201; 0.582) |  | 60 | 0.172 | (-0.409 ; 0.399) |
| 80 | 0.360 | (-0.487; 1.206) |  | 80 | 0.792 | (-0.582 ; 1.146) |

**Supplementary Figure S4.** Sensitivity analysis for the dose-response meta-analysis: exclusion of Modabbernia et al. for negative symptoms (n=8) and positive symptoms (n=6).

In this sensitivity analysis, we excluded the outlier study of Modabbernia et al..

For positive symptoms, the trend-level effect significance found prior to exclusion of this study dissipated.

For negative and positive symptoms, both effect sizes were nonsignificant (SMD, 0.011; 95% CI, -0.03 to +0.05; p=0.62; and; SMD, 0.03; 95% CI, -0.015 to +0.07; p=0.27; respectively).
