# Supplementary material for: Intranasal Oxytocin for Negative Symptoms of Schizophrenia: Systematic Review, Meta-Analysis, and Dose-Response Meta-Analysis of Randomized Controlled Trials
Source: Int J Neuropsychopharmacol. 2021 Apr 23;24(8):601–14. doi: 10.1093/ijnp/pyab020 (PMC8378078; doi:10.1093/ijnp/pyab020)
Supplement: pyab020_suppl_Supplementary_Table_S1 [file pyab020_suppl_supplementary_table_s1.docx]

**Supplementary Table S1.** Risk of bias assessment for included RCTs

| **Study**  **(author, year)** | **Random sequence generation (selection bias)** | **Allocation concealment (selection bias)** | **Blinding of participants and personnel (performance bias)** | **Blinding of outcome assessment (detection bias)** | **Incomplete outcome data (attrition bias)** | **Selective reporting (reporting bias)** | **Other potential biases** |
| --- | --- | --- | --- | --- | --- | --- | --- |
| **1-Cacciotti et al. 2015** | Low risk | Low risk | Low risk | Low risk | Low risk | Low risk | - |
| **2- Lee et al. 2013** | Unclear | Low risk | Low risk | Low risk | Low risk | Low risk | - |
| **3-Gibson et al. 2014** | Unclear | Unclear | Low risk | Low risk | Low risk | Low risk | - |
| **4-Jarskorg et al. 2017** | Low risk | Unclear | Unclear | Unclear | Low risk | Low risk | - |
| **5-Buchanan et al. 2017** | Low risk | Low risk | Unclear | Low risk | Low risk | Low risk | - |
| **6-Dagani et al. 2016** | Low risk | Low risk | Low risk | Low risk | Low risk | Low risk | - |
| **7-Feifel et al. 2010** | Low risk | Low risk | Low risk | Low risk | Low risk | Low risk | - |
| **8- Davis et al. 2014** | Unclear | Unclear | Unclear | Unclear | Low risk | Low risk | - |
| **9-Modabbernia et al. 2014** | Low risk | Low risk | Low risk | Low risk | Low risk | Low risk | - |
